# Posterior Reversible Encephalopathy Syndrome Leading to Diagnosis of Acute Postinfectious Glomerulonephritis in a Pediatric Patient: A Case Report

**DOI:** 10.1155/crpe/9946572

**Published:** 2025-07-02

**Authors:** Samuel B. Hayworth, Desalyn L. Johnson, Erinn O. Schmit

**Affiliations:** ^1^Department of Pediatrics, University of Alabama at Birmingham Heersink School of Medicine, Birmingham, Alabama, USA; ^2^Department of Pediatrics, Cincinnati Children's Hospital Medical Center, Cincinnati, Ohio, USA

**Keywords:** acute postinfectious glomerulonephritis (PIGN), computed tomography (CT) venogram, magnetic resonance imaging (MRI) T2-FLAIR, pediatrics, posterior reversible encephalopathy syndrome (PRES), seizure

## Abstract

**History:** An 8-year-old previously healthy female presented to the emergency department after new-onset seizure activity. Three days prior, she experienced severe headaches and rash over her left lower extremity. She developed nonbilious, nonbloody emesis on the day prior to admission. The morning of presentation, she was found unresponsive, exhibiting right gaze deviation and clenched jaw concerning for seizure activity. Further history revealed recent Group A streptococcal pharyngitis, which had been treated with a 10-day regimen of amoxicillin. During this time, her mother reported decreased oral intake but normal urinary output, without dysuria or hematuria.

**Physical Examination:** Vital signs were notable for temperature of 101.7 degrees Fahrenheit, heart rate of 100 beats per minute, blood pressure of 167/97, respiratory rate of 28, and oxygen saturation of 96% on room air. The patient was drowsy but arousable. Her neck was supple without evidence of meningismus. A skin exam revealed an erythematous, crusting rash, resembling contact dermatitis on the left lower extremity below the knee. The patient was somnolent but oriented to self and able to follow simple commands. Cranial nerves II–XII were intact bilaterally. Sensory, motor, and coordination were unremarkable.

**Laboratory, Imaging, and Procedures:** Laboratory findings included leukocytosis, microscopic hematuria, decreased C3 and C4 levels, and positive antistreptolysin O (ASO) titers. A lumbar puncture was conducted with unremarkable cerebrospinal fluid (CSF) indices. Imaging revealed hypoattenuation in the occipital lobes on computed tomography (CT) venogram and hyperintensities in bilateral occipital lobes on magnetic resonance imaging (MRI) T2 fluid-attenuated inversion recovery (FLAIR) sequences, consistent with posterior reversible encephalopathy syndrome (PRES).

**Discussion:** PRES in pediatric patients has been associated with a variety of conditions including hypertension (idiopathic or secondary), renal disorders, autoimmune disorders, and hematologic or oncologic conditions. However, PRES secondary to acute postinfectious glomerulonephritis (PIGN) is rare. Hematuria on the urinalysis led to obtaining complement levels, and further elucidation of history helped to narrow the differential to PIGN due to streptococcal infection with confirmatory positive ASO antibody titer. This case highlights a rare sequala of a commonly seen pediatric infection.

## 1. Introduction

Posterior reversible encephalopathy syndrome (PRES) is an acute neurological condition that is characterized clinically by encephalopathy, headaches, seizures, and hypertension, with classic magnetic resonance imaging (MRI) findings of T2-fluid-attenuating inversion recovery (FLAIR) hyperintensities in the parietal and occipital lobes [1]. This case report presents an uncommon instance of PRES in an 8-year-old female, secondary to acute postinfectious glomerulonephritis (PIGN).

## 2. Case Presentation

An 8-year-old previously healthy female presented to the emergency department after new-onset seizure activity. Three days prior to presentation, she experienced severe headaches and rash over her left lower extremity. She developed nonbilious, nonbloody emesis on the day prior to admission. The morning of presentation, she was found unresponsive, exhibiting right gaze deviation and clenched jaw, concerning for seizure activity. Further history revealed recent Group A streptococcal pharyngitis, which was treated with a 10-day regimen of amoxicillin. During this time, her mother reported decreased oral intake but normal urinary output, without dysuria or hematuria.

Vital signs were notable for a temperature of 101.7 degrees Fahrenheit, heart rate of 100 beats per minute, blood pressure of 167/97, respiratory rate of 28, and oxygen saturation of 96% on room air. The patient was drowsy but arousable. Her neck was supple without evidence of meningismus. A skin exam revealed an erythematous, crusting rash, resembling contact dermatitis on the left lower extremity below the knee. Cardiac and abdominal examinations were unremarkable. A neurological exam revealed a Glasgow Coma Scale (GCS) of 14, with an eye subscore of 4, verbal subscore of 4, and motor subscore of 6. The patient was somnolent but oriented to self and able to follow simple commands. Cranial nerves II–XII were intact bilaterally. Sensory, motor, and coordination were unremarkable.

Laboratory investigations revealed an elevated white blood cell (WBC) count of 30.77 × 10^9^/L and a C-reactive protein (CRP) level of 0.17 mg/dL. A comprehensive metabolic panel (CMP) showed normal serum electrolytes (Na^+^ 137 mmol/L, K^+^ 4 mmol/L, and Cl^−^ 106 mmol/L), slightly decreased bicarbonate (CO_2_^−^ 21 mmol/L), and normal renal function (Cr 0.42 mg/dL and BUN 10 mg/dL). Lumbar puncture revealed normal cerebrospinal fluid (CSF) indices. Blood cultures were obtained, and empiric intravenous antibiotics were initiated. She was then admitted to the pediatric intensive care unit (PICU).

**Figure 1:**
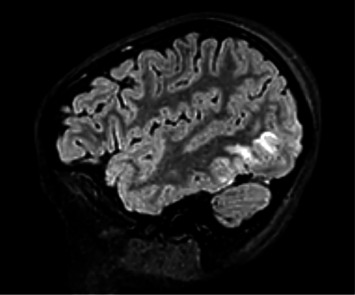
Sagittal MRI T2-FLAIR sequence displaying vertical hyperintensities in the posterior aspect of the left temporal lobe.

**Figure 2:**
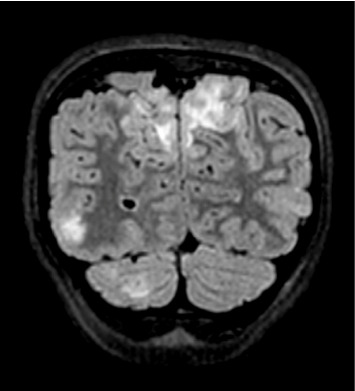
Coronal MRI T2-FLAIR sequence with hyperintensities in bilateral parietal–occipital lobes, right inferior occipital lobe, and right cerebellar hemisphere.

A computed tomography (CT) venogram of the head with and without contrast revealed hypoattenuation in bilateral paramedian occipital lobes. Brain MRI with T2-FLAIR sequences showed vertical hyperintensities in the posterior left temporal lobe (Figure 1), as well as hyperintensities in the right cerebellar hemisphere and bilateral occipital lobes (Figure 2), highly consistent with PRES.

In the PICU, her hypertension was rapidly controlled with antihypertensive medications, leading to normotensive readings and improvement in mental status. She was then subsequently transferred to the pediatric hospital medicine service. At that time, urinalysis revealed hematuria without proteinuria, with 3+ blood and 37 erythrocytes per high-powered field (HPF). Complement levels showed markedly decreased C3 (< 11 mg/dL; normal: 83–152 mg/dL) and low C4 (9.5 mg/dL; normal: 13–37 mg/dL). Antistreptolysin O (ASO) titers were significantly elevated at 1446 IU/mL (normal: < 250 IU/mL). These findings, along with a recent history of streptococcal pharyngitis, elevated PIGN as the most likely etiology. In addition, an antinuclear antibody (ANA) panel was negative, helping to exclude lupus nephritis, C3 glomerulopathy, and membranoproliferative glomerulonephritis (MPGN). Renal ultrasound was unremarkable. Our team subsequently diagnosed the patient with acute PIGN, with PRES as a sequela of rapid-onset hypertension.

As her blood pressure improved, the patient's symptoms gradually resolved. Her headache was minimal, and her mental status returned to the baseline. Vital signs remained stable and within normal limits, with normal oral intake and urine output. After 5 days of hospitalization, she was discharged home with a prescription for amlodipine and advised to follow up as an outpatient. At 6-week follow-up, C3 levels improved to 70 mg/dL. Repeat MRI was not obtained due to the need for sedation.

## 3. Discussion

The leading diagnosis of PRES as a sequela of PIGN was determined by a complete assessment of the clinical presentation, laboratory, and radiological data. New-onset seizures and sudden hypertension pointed to hypertensive encephalopathy and PRES, with the underlying cause of hypertension initially unclear. Urinalysis and recent history of group A streptococcal pharyngitis supported PIGN as the primary cause. PRES commonly presents with symptoms of acutely increased intracranial pressure, with symptoms of headache, nausea, vomiting, seizures, altered mental status, and visual changes. These symptoms result from vasogenic edema, typically involving the occipital and parietal white matter [2]. Reported triggers include preeclampsia, eclampsia, renal failure, sepsis, thrombotic thrombocytopenic purpura, hemolytic uremic syndrome, vasculitis, porphyria, and lupus. In addition, PRES has been linked to side effects of antineoplastic drugs and immunosuppressant agents, including cisplatin, cyclosporine, and cyclophosphamide [3, 4].

In our patient, the initial blood pressure reading of 167/97 mmHg represented a severe elevation and met the criteria for stage 2 hypertension based on the 2017 American Academy of Pediatrics Clinical Practice Guideline for Screening and Management of High Blood Pressure in Children and Adolescents [5]. This degree of hypertension likely played a central role in the development of PRES, as hypertensive crises are a well-established trigger. The exact pathophysiology of PRES remains incompletely understood, but hypertension and endothelial injury seem to be present in the majority of cases. Sudden elevations in systemic blood pressure can exceed the autoregulatory capacity of the brain vasculature, leading to blood–brain barrier disruption and fluid extravasation [3, 4]. Acute, sustained increases in mean arterial pressure (MAP) of more than 150–160 mmHg disrupt these autoregulation mechanisms and cause hyperperfusion, cerebral vessel damage, and interstitial extravasation of proteins and fluid, causing vasogenic edema. Although this theory remains the most accepted, it does not explain the development of PRES in normotensive patients or patients with only mildly elevated blood pressure. Approximately 20%–30% of the patients present with normal blood pressure, reinforcing the notion that the pathophysiology of PRES is multifactorial and not yet fully understood [3, 6].

The development of PRES in the pediatric population is rare but increasingly recognized [7]. Although typically reversible, PRES has been associated with worse neurological outcomes in children. In critically ill pediatric patients matched for age and severity of underlying disease, those with PRES have higher mortality [8]. Children are hypothesized to have a narrower range of cerebral autoregulation, increasing their risk of PRES during acute rises in systemic blood pressure. In nonneoplastic-related PRES, renal etiologies are a common cause in pediatric cases. A 2022 systematic review evaluated 449 noncancer pediatric PRES cases. Of these, 165 (36%) were due to kidney disease, with PIGN accounting for just 20 cases (4%). Hypertension is a common presenting finding in pediatric PIGN, and 3%–6% of these patients will go on to develop long-standing hypertension [9, 10].

MRI typically shows cortical and subcortical vasogenic edema in the parietal and occipital lobes and appears as hyperintense signals on T2-FLAIR sequences. Other common sites of vasogenic edema include the frontal lobes, the inferoposterior temporal lobes, and cerebellum [3, 11]. MRI findings in PRES are usually reversible on follow-up and correlate with clinical improvement [11]. Diffusion-weighted imaging (DWI) distinguishes vasogenic from cytotoxic edema: vasogenic edema shows increased diffusion due to interstitial water, while cytotoxic edema shows markedly decreased diffusion [12]. Therefore, DWI is the modality of choice to confirm PRES [3].

Prompt recognition and management of precipitating factors are crucial to reversing PRES [13]. No randomized trials have assessed therapeutic interventions for PRES; thus, no specific treatment is available. Supportive, symptom-directed management is imperative and typically consists of antihypertensive and anticonvulsant agents, removal of inciting agents, and correction of comorbidities driving hypertension. Aggressive blood pressure control is essential but must be achieved through gradual MAP reduction to avoid organ hypoperfusion. Patients with severe PRES manifestations (e.g., status epilepticus, coma, respiratory failure, or hypertensive crisis) may require intensive care [1]. Timely treatment usually results in full recovery within days to weeks [3].

## 4. Conclusion

PRES should be considered in patients presenting with sudden encephalopathy, even in the pediatric population where it is less common. This case highlights an unusual sequela of a frequently encountered pediatric infection. MRI, particularly T2-FLAIR and DWI sequences, is essential in confirming the diagnosis. Although no specific treatment exists, timely intervention generally yields favorable outcomes, highlighting the importance of prompt recognition and understanding of PRES in clinical practice.

## Data Availability

Data sharing not applicable to this article as no datasets were generated or analyzed during the current study.
